# Boils, Carbuncles, and Whitlows

**Published:** 1897-10-16

**Authors:** 


### BOILS, CARBUNCLES, AND WHITLOWS.

Amid the rush and urgency of surgical work it is perhaps a relief, it is at any rate a change, to turn aside into the easy and oleaginous paths of the physician who treats boils, carbuncles, and whitlows by means of ointments and internal medication.

Speaking at Montreal, Dr. Duncan Bulkley said that for ten years he had avoided the use of both the poultice and the knife in treating these affections. The first point in dealing with them is, according to Dr. Bulkley, the treatment of the constitutional defect which underlies all these ailments. He begins with a mercurial purge of blue pill, colocynth, and ipecacuanha. Two pills at night, and two on the second night after. These four pills are generally repeated at the end of a week, and perhaps in other successive weeks. A mixture of sulphate of iron, sulphate of magnesia, and sulphuric acid is also to be given after meals.

To boils the following ointment is applied: Carbolic acid, grains 5 to 10; fluid extract of ergot, 1 to 2 drachms; powdered starch, 2 drachms; oxide of zinc, 2 drachms; rose-water ointment, 1 ounce. A considerable mass of it being spread on an oblong piece of absorbent cotton, this is laid over the boil, where it is kept in place by strips of adhesive plaster across the ends of the piece, but not over the boil itself. If comfortable, this dressing is left untouched for 12 or more hours, when it is removed, and a freshly-spread piece immediately re-applied. If there has been any discharge, the surface may be very gently cleansed with absorbent cotton, but there must be no squeezing. In many instances the boil aborts, but when this does not happen it ruptures spontaneously in a relatively short time. The treatment is applicable to all forms of boils, and the ointment is to be continued until they either abort or are quite healed.

Carbuncles are to be treated in the same way. In some cases febrile symptoms may occur which may seem to warrant more active interference with the knife, but both in regard to time, final results, pain, and general comfort, the ointment treatment is held to be far superior. Sometimes a little squeezing may be required, or the use of forceps to hasten the separation of sloughs.

In the treatment of whitlow, Dr. Bulkley uses the diachylon ointment prepared according to the formula of Hebra. This ointment is of a soft, buttery consistence, and the finger is to be covered with a thick layer of it, completely enveloping the first joint to a thickness of a quarter of an inch. Over this are placed layers of absorbent cotton, and the whole is loosely bound up. This treatment is to be applied not only to whitlows of a superficial character about the nails but also to those which are very deeply seated on the pulp of the finger, even when there have already been sleepness nights from the throbbing.

It is a good thing, perhaps, to be reminded now and again that the quick elimination of septic matter is not the beginning and the end of all therapeutic effort, that such diseases as these will sometimes get well without the knife, and that they do not of necessity produce the evil consequences which are so frequently found in neglected cases; and it is certainly interesting to know what line of treatment is found most useful by a physician who has definitely laid down for himself the task of dealing with such things without the knife. Moreover, the whole affair seems delightfully simple, and perhaps, in regard to boils, no harm may be done if it misses fire. It is not safe, however, to be too hopeful about whitlows.

